# Canonical mRNA is the exception, rather than the rule

**DOI:** 10.1186/s13059-017-1268-1

**Published:** 2017-07-07

**Authors:** Jun-An Chen, Simon Conn

**Affiliations:** 1 Institute of Molecular Biology, Academia Sinica, Taipei, 11529 Taiwan; 2 Centre for Cancer Biology, an alliance between SA Pathology and University of South Australia, Adelaide, SA 5000 Australia

**Keywords:** Non-coding RNA, Review, Circular RNA, lncRNA, miRNA

## Abstract

A report on the Second Aegean International Conference on the Long and the Short of Non-Coding RNAs, held in Heraklion, Greece, 9–14 June 2017.

## Introduction

Investigations into gene regulation and disease pathogenesis have been protein-centric for decades. However, in recent years there has been a profound expansion in our knowledge of the variety and complexity of eukaryotic RNA species, particularly the non-coding RNA families. Vast amounts of RNA sequencing data generated from various library preparation methods have revealed these non-coding RNA species to be unequivocally more abundant than canonical mRNA species. Furthermore, insight into the diverse mechanisms and functional roles of these RNA transcripts is emerging, pointing to their roles in maintaining cellular homeostasis and gene regulation and/or directing various pathologies.

The Second Aegean International Conference on the Long and the Short of Non-Coding RNAs focused on the key contemporary findings in this field. Organised by a scientific committee comprising Judy Lieberman (Harvard Medical School, Boston, USA), Zissimos Mourelatos (University of Pennsylvania, Philadelphia, USA) and Andrei Thomas-Tikhonenko (The Children's Hospital of Philadelphia, Philadelphia, USA), the conference was held at the Aldemar Knossos Royal Village Conference Center in Heraklion, Crete (Greece), 9–14 June 2017, and was attended by 125 delegates from numerous countries. There were no defined keynote talks, but 25 invited talks and 15 short contributed talks, covering a breadth of small and long non-coding RNA species from yeast, *Drosophila*, plants and mammals, even presenting the therapeutic potential of these molecules in various diseases. Plenary talks were further complemented by active poster sessions, where participants engaged with more than 85 poster presenters during conference breaks.

In this report, we briefly recount the conference content by providing condensed, headline-style summaries of the research described in the talks and some of the posters. Within the scope of this brief report we have highlighted three key research themes: non-coding RNAs in disease; circular RNAs and their formation and function; and RNA stability. However, we cannot possibly do justice to the quality and quantity of all work presented. We would like to stress that omissions from this report are not based on quality, but simply a personal judgement as to which material could be most coherently presented within limited space.

## Non-coding RNAs in disease

Perhaps the most pivotal discovery regarding non-coding RNAs are that they are not simply transcriptional by-products, or splicing artefacts, but comprise a diverse population of actively synthesised and regulated RNA transcripts. These transcripts can—and do—function within the contexts of cellular homeostasis and human pathogenesis. Technologies based on next-generation sequencing have revealed many novel classes of non-coding RNA transcripts, but biochemical characterisation to ascribe the mechanisms behind their functions, especially in vivo, provides an essential roadmap to guide the research field. Two overriding questions remain to be answered: 1) how is long non-coding RNA (lncRNA) targeted to the genome, and 2) are lncRNAs functionally important?

Ingrid Grummt (German Cancer Research Center, Heidelberg, Germany) elegantly demonstrated that an E2F1-regulated antisense lncRNA named *Khps1* activates expression of its cognate mRNA, *SPHK1* (*sphingosine kinase 1*). This is achieved through forming a DNA–RNA triplex that recruits histone acetyltransferase p300/CBP and other associated effector proteins to the gene promoter. By developing a biochemical purification method for DNA–RNA triplexes as an alternative to ChiRP-seq (chromatin isolation by RNA purification), the Grummt group proposed a hotly debated model that these triplexes might be a general characteristic of target gene recognition by chromatin-interacting lncRNAs. While genome-wide binding sites for MEG3 lncRNA supported this new model, Jun-An Chen’s group (Academia Sinica, Taipei, Taiwan) found the same lncRNA to be highly expressed in motor neurons, following systematic profiling studies on embryonic motor neuron-enriched lncRNAs. Loss of *Meg3* directs motor neuron subtype identity by regulating the epigenetic landscape through PRC2, resulting in altered expression of homeotic genes and homeotic transformation in knockout mice. Martin Crespi (Institute of Plant Sciences Paris-Saclay, Paris, France) reported another homeotic transformation effect for a lncRNA in *Arabidopsis*, whereby broad modulation of alternative splicing resulted in altered root branching. Together, these reports endorse lncRNAs as having a function in homeotic transformation.

Iannis Aifantis (NYU Langone Medical Center, New York City, USA) performed a genome-wide lncRNA reverse-genetics screen with an enhanced Cas9 repressor protein to investigate regulation of key targets downstream of Myc. He identified a conserved lncRNA that coordinated chromosome conformation (three-dimensional DNA looping) in T-cell acute lymphoblastic leukaemia, with knockdown studies reducing tumours in vivo.

These systematic analyses were also employed by other researchers at the meeting, including Andrea Ventura (Memorial Sloan Kettering Cancer Center, New York, USA), whose talk “Cancer Modeling in the CRISPR Age” reaffirmed this approach as the gold-standard for genetic screens in cell lines and model organisms alike. Joshua Mendell (UT Southwestern Medical Center, Dallas, USA) reported on a Cas9 genome-wide loss-of-function screen to identify new regulators of the microRNA (miRNA) pathway. This screen revealed a novel phosphorylation and dephosphorylation cycle for Argonaute 2 (AGO2), which is stimulated by regulated interactions between miRNA and targets to maintain the global efficiency of miRNA-mediated silencing. Collaborative distribution of the outcomes of these CRISPR screens might greatly accelerate research outputs, and was strongly encouraged by meeting delegates.

In concatenated talks, Jean-Christophe Marine and Eleonora Leucci (both from VIB-KU Leuven Center for Cancer Biology, Leuven, Belgium) narrated an elegant study on a nearly ubiquitous, melanoma-specific oncogene (MITF)-related lncRNA, *SAMMSON*. They demonstrated that the “addiction of melanoma” to this lncRNA is mediated through its interaction with distinct proteins, with contrasting functions, to regulate melanoma progression. This finding prompts an interesting hypothesis that lncRNA might function as a molecular switch to rewire RNA binding proteins in cancer.

Multiple high-profile presenters, including David Bartel (Massachusetts Institute of Technology, Cambridge, USA) and David Corey (UT Southwestern Medical Center, Dallas, USA), thematically questioned whether ncRNA copy numbers were sufficiently abundant to co-regulate target molecules (whether protein, RNA or DNA) in a physiological context. Corey further encouraged all attendees to assess absolute abundance of focal ncRNA and their target(s) and also their subcellular localisation—an approach that contributed greatly to his own study on factors involved in Argonaute-mediated nuclear RNAi. Quantifying the stoichiometric relationships between ncRNAs and their targets will likely be necessary in future research reports.

## Circular RNAs: formation and function

As “new kids on the block” in the non-coding RNA family, the circular RNAs (circRNAs) are a large group of uniquely alternatively spliced RNA molecules, which are ubiquitous among eukaryotes and are proving functional in a plethora of contexts. Historically much maligned because of their relatively low abundance and high diversity, it may be surprising that five individuals’ presentations focused predominantly or exclusively on discrete aspects of circRNA biogenesis, recognition and regulation. Some presented on circRNA functions hitherto unheard-of for any ncRNA.

Mariangela Morlando (Universita di Roma, Rome, Italy) showed that, while not widespread, certain circRNAs are not strictly non-coding RNAs at all. An example being circ-ZNF609, these circRNAs retain endogenous internal ribosome entry sites from the cognate mRNA, which enables their translation into protein. She also identified a new circRNA biogenesis factor, FUS, whose pathogenic mutations in amyotrophic lateral sclerosis and autism are linked to altered circRNA formation. Gregory Goodall (Centre for Cancer Biology, Adelaide, Australia) reported on the role of Quaking, an RNA binding protein that regulates circRNA biogenesis in epithelial–mesenchymal transition, a model of cancer metastasis. Nikolaus Rajewsky (Max Delbrück Center for Molecular Medicine, Berlin, Germany) described how CRISPR-Cas9-based genome editing could be used to remove the expression of one brain-specific circRNA, CDR1as. This is a ‘sponge’ for human microRNA-7 (hsamiR-7), which yields mice with a normal phenotype but mildly altered neurological responses.

Simon Conn (Centre for Cancer Biology, Adelaide, Australia) reported another seminal finding by demonstrating that circRNAs are stoichiometrically favoured to bind certain targets in the cell. Specifically, interactions between a circRNA from *Arabidopsis* and its cognate DNA locus via complementary base-pairing to produce an RNA:DNA hybrid, or R-loop, is sufficient to promote alternative splicing of its cognate mRNA, altering floral morphology. The capacity of circRNAs to bind DNA was supported by Ingrid Grummt, who reported that circRNAs, as well as other ncRNAs and linear RNAs, associate with DNA without complementary base-pairing in DNA–RNA triplexes. Yet, the hyperstability of circRNAs means they are potent mediators in any landscape, particularly in dynamic chromatin.

It has long been recognised that single-stranded RNA viruses, recognised as foreign entities, can drive host immune responses. However, the fact that circRNAs are structurally identical led opening speaker Howard Chang (Stanford University, Stanford, USA) to reason that circRNAs might do the same. As shown by ChiRP–MS (comprehensive identification of RNA binding proteins by mass spectrometry), recognition of self versus non-self circRNAs depends on their biogenesis and marking by endogenous protein co-factors. Importantly, the use of circRNAs seems as effective as current metal ion adjuvants in promoting immune responses to vaccination and to block viral transduction.

Taken together, we have learned much about circRNAs since their systematic identification by NGS in 2013. It is increasingly clear that they are not accidental by-products of splicing. The talks summarized above provide insights into the ways in which circRNAs are involved in neurodevelopment and degeneration, plant development, and cancer. Studies of how circRNA is generated and its functions at the organismal level will surely proceed rapidly in the coming years.

## RNA stability

The functional consequence of miRNA binding to mRNA was once considered a one-way street, whereby miRNAs induce mRNA target instability. However, the reverse situation, called target-induced miRNA degradation (TIMD), is now emerging as a physiologically relevant phenomenon. Although miRNA biogenesis is well characterised, its regulation and the mechanism underlying miRNA turnover are not yet entirely understood.

Both Paulina Pawlica from Joan Steitz’s group (Yale University, New Haven, USA) and Francesco Nicassio (Istituto Italiano di Technologia, Genova, Italy) presented data on TIMD, including a proposed general mechanism for miRNA-degradation elements: the miRNA-binding region may not only be complementary to the 3′ end of miRNA, but with conformational flexibility can efficiently mediate miRNA decay. Nikolaus Rajewsky demonstrated that loss of a circRNA that acts as an miRNA ‘sponge’ also destabilises its miRNA and deregulates expression of its target mRNAs. Lynne Maquat (University of Rochester Medical Center, Rochester, USA) also revealed a new pathway for miRNA decay: functional miRNAs are degraded in human cells by the endonuclease Tudor-SN, and this is functionally critical for cell cycle progression. David Bartel found that RNA secondary structures are more prevalent within extended 3′-end regions of poly(A) signals than upstream regions. Experiments have demonstrated that this folding both enhances processing and increases mRNA metabolic stability, adding another possible layer of complexity to the model that miRNAs can target non-contiguous, flanking sequences of these structured regions.

The myriad functions of miRNA have been well illustrated over the past decade, yet we are in a renaissance age of studying miRNA functions in animal models. Several miRNA knockout studies and salient phenotypes were revealed at this meeting. Robert Blelloch (University of California, San Francisco, USA) posed the question as to why some evolutionarily conserved miRNAs have the same seed sequences and similar expression patterns, yet manifest distinct phenotypes. His findings suggest that the same-seed miRNA family share redundant and non-redundant roles. In agreement, Lin He (University of California, Berkeley, USA) showed that knockouts of other compound mutants of the same-seed miRNA cluster family showed redundancy, until complete knockout was achieved. Taken together, these knockout analyses of miRNAs and lncRNAs indicate that ncRNAs are certainly important in embryonic development and disease progression. As miRNA and lncRNA knockout mice become increasingly available, perhaps we will learn that ncRNAs function in organ development and immunity, and link them to human diseases.

Although many more interesting topics were discussed at the Crete meeting, including covalent RNA modifications, epitranscriptomics (Chuan He, Richard Gregory, Judy Liebermann) and regulated patterns of RNA cleavage/polyadenylation (Zissimos Mourelatos, Christine Mayr, Martine Simonelig), these could not be covered here.

## Headline new technologies and applications

Lin He: novel methodology for efficient, cost effective CRISPR-targeted mice, called CRISPR-EZ.

Zissimos Mourelatos: co-translational degradation of engaged mRNAs (ribothripsis).

Nikolaus Rajewksy: droplet-seq to digitally reconstruct, cell-by-cell, the *Drosophila* embryo gene expression profile.

## Conclusions and outlook

The first long and short ncRNA meeting took place in Crete two years ago, bringing together ncRNA biologists from a narrow range of disciplines. Since then, our understanding of the molecular basis of the key processes underlying ncRNA regulation and their implications for disease and development has considerably increased. We now understand more about the molecular cues that direct the production of circRNAs, and their functions are emerging. Complementing these advances is our progress in understanding the mechanisms regulating long and short ncRNAs. The first and second meetings on ncRNAs in Crete will accelerate developments in the field of ncRNA biology by bringing together a more interdisciplinary field of scientists and, consequently, more systematic analyses. We enthusiastically await and can only imagine the advances to be presented at the third meeting in 2019.

## Abbreviations

Cas9: CRISPR-associated protein 9
circRNA: Circular RNA
CRISPR: Clustered regularly interspaced short palindromic repeats
lncRNA: Long non-coding RNA
miRNA: MicroRNA
ncRNA: Non-coding RNA

